# Enhancing your practice: debriefing in interventional radiology

**DOI:** 10.1186/s42155-023-00412-8

**Published:** 2024-01-16

**Authors:** Kara Fitzgerald, Jesse Knight, Karim Valji

**Affiliations:** Department of Radiology, Section of Interventional Radiology, University of Washington, Seattle, Washington, USA

**Keywords:** Debriefing, Teamwork, Communication, Interventional radiology, Second victim, Burnout, Quality improvement

## Abstract

**Learning objectives:**

Review the history of debriefing and provide an Interventional Radiologist (IR) specific framework for leading an effective debrief.

**Background:**

A debrief is often regarded as a meeting with persons who were involved in a stressful, traumatic and/or emotionally challenging situation to review processes, communicate concerns or gather feedback. The goals of these sessions can be for learning/quality improvement (QI) or psychological/emotional support, or a mix of both. Debriefing after tough situations has become a standard tool of many medical specialties, such as surgery, critical care and emergency medicine, with specialty specific literature available. However, there is a paucity of Interventional Radiology specific literature available for debriefing techniques.

**Clinical findings/procedure details:**

We will review the history and types of debriefing and why a debrief could be considered. We will provide a framework for leading a successful debrief in Interventional Radiology.

**Conclusion:**

Debriefing can be a useful tool for learning and QI as well as psychological or emotional support after a challenging or tough situation. Debriefing can address multiple variables and can stylistically be tailored to suit specific needs. IRs have an opportunity to take a leadership role in debriefing, providing comfort and quality improvement through communication and support.

## Background

### What is a debrief

When caring for patients, an Interventional Radiologist (IR) can face stressful, traumatic and/or emotionally challenging situations which often coincide with an adverse event (AE), critical incidents (eg., unexpected patient death or mass casualty), near misses of these events or dysfunctional interpersonal interactions with colleagues, staff or other physicians. For the purpose of this article, these aforementioned scenarios will be referred to as “tough situations.” These tough situations can be mentally perplexing, with no clear path forward at times.

The term debriefing has a relatively broad connotation. According is an intentional discussion used to answer specific questions about a patient event, for the use of knowledge or skill attainment, for therapeutic intervention or for making improvement in performance [1]. In some medical specialties, debriefing has been applied more broadly, independent of the perceived challenge. For example, routine use of debriefing after every surgery prior to the departure of the staff/surgeon from the operating room or recurring debriefs on a unit or ward as part of daily or weekly routine. Some facilities will provide robust structured debriefing led by someone with specific training for leading debriefs after a catastrophic event such as mass casualty, which has been referred to as critical incident debriefing [2, 3]. The definition of debriefing for this paper refers to discussion between persons involved in tough situations with the goals of reviewing processes, communicating concerns, providing emotional support, collecting feedback or identifying opportunities for quality improvement (QI).

There are many reported styles of clinical debriefing. In a scoping systematic review of debriefs in medicine by Evans et al., there were two general themes of debriefing found [2]. The first theme is discussion of the event proceedings for learning or QI and second is for supporting individuals after experiencing a traumatic/adverse event. Examples of the first category (learning and QI) include REFLECT (Review the event, Emphasize key points, Communicate clearly, Transform the future), TALK® (Target, Analysis, Learning, Key action) and PEARLS (The Promoting Excellence and Reflective Learning in Simulation) [2]. An additional learning and QI model described by Zigmont et al. is the 3D Model of Debriefing [4]. Examples of the latter theme (psychological or emotional support) includes the Critical Incident Stress Debriefing (CISD) model and Psychological Debriefing and Trauma Risk Management [2].

The debriefing process should be thought of separately from a root cause analysis process, although findings or concerns brought up in a debrief can be used to initiate or contribute to a root cause analysis [1]. Debriefing is also in a slightly different process from defusing, where the goal is to vent and reduce tensions [5].

This paper will review the history of debriefing, explore potential benefits of debriefing in clinical practice, address the concept of a second victim, and give IRs a framework to lead a team through a debrief.

### History of debriefing

Formal debriefing for learning or QI was first employed in military operations (World War II era) before being used in other critical fields including aviation and later medicine [5, 6]. A system developed by the United States Army in the 1970s called the After Action Review (AAR) was defined as “a professional discussion of an event, focused on performance standards, that enables soldiers to discover for themselves what happened, why it happened, and how to sustain strengths and improve weaknesses” [7]. There were two specific experiences the Arm drew on for development of the AAR. First was interviewing soldiers. This was done by World War II Army journalists/historians who were working to actively and accurately document the proceedings of the War, and would interview soldiers immediately after battle. Second, was the Army’s prior technique of performance critique. This previously used style was a subjective process review directed by senior leaders in lecture format and typically had a negative tone. Although it was used for years, this style was felt to be counterproductive to unit performance. Drawing from these experiences the AAR was developed, where the leader acted as a facilitator asking open ended questions, allowing participants to provide objective information and critique their own performance.

AAR sessions are held after events in combat or training exercises, including simulation. The general question is “How did the unit do?” [7]. This is further broken down into more directed questions: 1. “What happened during the collective training exercise?” 2. “Why did it happen?” 3. “How can units improve their performance?” [7]. An AAR session could be “informal” lasting 15 to 30 minutes and occurring directly after an event, or “formal” lasting 90 to 120 minutes and occurring farther from the event, with substantial preparation before the session [7]. The AAR technique became popular and was translated to civilian use, particularly in the corporate arena, using similar techniques to review meetings and projects.

The CISD model for psychological debriefing after a “critical incident” was developed in the 1970s–1980s by psychologist Dr. Jeffrey Mitchell, PhD. These “critical incidents” were “work trauma” events and defined as “any situation faced by emergency service personnel which overwhelms the usual coping strategies and has the capacity to interfere with their ability to function either at the scene or later” [3, 8]. Prior to creating the CISD model, Mitchell had worked as a first responder. CISD was initially created for first responders, as they can experience high levels of stress, be exposed to death and suffering and carry the responsibility of others’ lives and community wellbeing [8].

The CISD technique describes a structured approach amongst a homogenous cohort of first responders led by a formally trained person (typically mental health specialist) to discuss the event and explore the involved parties’ coping and psychological status [8]. This formal process lasts two to 3 hours. The goal is to lessen the impact of trauma and help these workers return to their normal function [8]. Literature has demonstrated decreased stress related symptoms when this model was used in a homogenous cohort of first responders [2, 8–10]. However, in some studied cohorts such as heterogeneous groups of non-first responders (eg. trauma victims), there is data to suggest negative psychological sequelae with increased psychological morbidity using the CISD method, thought to possibly be related to talking through the events [2, 11]. Overall, there is a paucity of robust data in medicine exploring the use of group based psychological debriefing after adverse events [2, 12].

A similar format to AAR was developed in medicine after a critical publication by the Institute of Medicine *To Err is Human* in 1999, which provoked a multi-agency focus on QI in patient care and teamwork [13]. The TeamSTEPPS™ model was initiated in 2003 through collaboration of the Department of Defense and the Agency for Healthcare Research and Quality [13]. This model is based on 25 years of research with a focus in human factors engineering, human error and medical team training [13]. The framework of TeamSTEPPS™ is understanding the relationship of knowledge, attitudes and performance of a team and individuals and how this relates to leadership, communication, situational monitoring and mutual support. Debriefing is described in part of their “tools and strategies” among other commonly employed techniques used across medicine such as hand offs and SBAR (Situation, Background, Assessment and Recommendation) communication.

In 2008, Salas et al. created a list of 12 evidence-based, best practice techniques to lead a debrief. Since then, multiple other approaches to debriefing have been developed and applied to an array of different groups or scenarios in medicine. One final model for discussion is the 3D Model of Debriefing: Defusing, Discovering and Deepening [4]. A key defining difference is the model is based in adult learning theory. The focus is on the Learning Outcomes Model, which includes the individual, the key experiences and the learning environment, which contributes to effective practice based learning [4].

Moreover, there are many debriefing techniques used in medical simulation training, which are beyond the scope of this paper. One model to point out specifically is the Plus-Delta model, which denotes benefits of simplicity and is strongly based in self-assessment, which is noted to be a “vital skill for safe clinical practice” [14].

### Why debrief

Patient care can benefit from debriefing. One prominent example was to incorporate debriefing after cardiac arrest. Wolfe and coauthors demonstrated improved survival with favorable neurologic outcomes when post event, interdisciplinary debriefing was used [12].

Physicians may also benefit from debriefing. A caregiver who has indirectly been harmed or experiences psychological distress after an AE has been referred to as a “second victim” [5, 15]. Physicians who experience AEs were more likely to report burnout [16]. Additionally, burnout associated with AEs was worse amongst physicians who did not have peer support [16]. Moral injury, stress and burnout can be seen as a result of ignored emotional wellbeing [2]. One recent qualitative study regarding general surgery resident perceptions for improved wellbeing was to incorporate debriefing sessions after challenging patient outcomes [17]. Debriefing can provide opportunity for emotional support and peer support after a tough situation, or identify additional resources available to the involved parties. There is no specialty specific literature related to debriefing for emotional support in interventional radiology despite IRs experiencing these types of tough situations.

Debriefing can improve team performance. In high acuity, high stress situations with ambiguous realtime information, medical teams are not inherently set up for optimal performance [6]. Debriefs can positively affect team performance by way of the shared understanding that is developed [6]. Additionally, debriefs can strengthen team trust and promote positive interactions between team members [6].

In a recent review article by Clements and Koukounaras discussing complications in Interventional Radiology, they comment that debriefing is an important step after an AE to identify and record processes which have failed and may benefit from review or improvement [18]. QI is a critical component of being an IR and an integral part of workflow to continue to advance the practice and safety of Interventional Radiology [19]. Similarly, there is a paucity of Interventional Radiology related literature related to debriefing for QI purposes.

There are long-standing techniques that have been used across medical specialties, corporations and military operations to use debriefing for QI and learning as well as emotional and psychological support. These techniques should not be avoided due to lack of specific data, rather they should be used to enhance the vibrant and growing field of Interventional Radiology and be researched along the way.

### How to debrief

It is paramount to create an emotionally or psychologically safe environment to conduct the debrief [1, 5, 6, 20]. Facilitating a comfortable, supportive environment for learning and sharing will provide the best opportunity to optimize engagement of the involved parties.

Kessler et al. notes there are multiple strategies that can be used in debriefing and the methods can be tailored to the clinical scenario [5]. Additionally, there is not one clear successful technique, rather general trends of components to include and to avoid. For these reasons, standardization can be difficult. Standardization in a specific care area could be beneficial so that team members could anticipate debriefing and increase frequency [5]. While the components to include should help reach clinical care goals, care of the individuals within a team is vital to success. It was also noted that formal training should be considered to optimize the impact of the sessions, however this can be at the discretion of a physician [13, 17].

Debriefing must be supported by hospital administration to ensure time is allocated and team members prioritize debriefing [6].

Based on The Joint Commission Journal on Quality and Patient Safety paper *Debriefing Medical Teams: 12 Evidence Based Best Practice and Tips* and other available literature, the authors have created a guide to debriefing that can be used by an IR.

## Guide to leading a debrief

### Situations to debrief

- Could be considered after any adverse event including unexpected patient death or catastrophic complication (e.g., paraplegia after bronchial artery embolization) significant medication errors, cardiac arrest and resuscitation efforts, patient death, care of a critically ill child, mass casualties.
- Can be used as a diagnostic work up for QI and learning or for psychological and emotional support, or both. Identifying the goal of the debrief will tailor the techniques or questions used.

### Who to include

- All care team members involved in the tough situation. On some occasions it could be more helpful to have a smaller debrief of similar staff position (e.g., physician, nurse, technologist) particularly for QI debriefs.
- Occasionally, individuals who were not directly involved but have support role (e.g., social worker or technologist / nurse manager) could be helpful for support or note keeping.

### Timing

- A debrief can be immediate (“hot”), shortly after (“warm”) or delayed (“cold”) depending upon the scenario and availability for attendance. If a catastrophic event is encountered, a hot or warm debrief may be preferred to have earlier support, which allows the opportunity to acknowledge the events, accept the outcome and identify additional areas of attention (e.g., QI components, additional support for those involved, feedback for managers).

### Creating a safe environment

- Choose a location and setting that is appropriate, quiet and relatively private.
- Professional, kind communication is imperative. Focus conversion on “I” or “we” rather than “you” statements.
- Care should be taken to engage in open ended questions, creating a comradely atmosphere.
- Tone, body language and communication style should be considered and of focus for maintaining a constructive session (e.g., all members either seated or standing to avoid subconscious power differential). Avoid crossing arms or defensive posturing.
- Speaking or sharing should not be mandatory.

### Ground rules

- Set expectations of overall time of the session and rough limits for individuals speaking.
- State the goal(s) of debrief amongst the participants.
- Objective data gathering is helpful for QI whereas subjective experiences may be more common in emotional support geared debriefs.
- Identify what will happen with feedback or action items identified.
- No Blame Zone: The goal of any debrief should be psychological safety and discovery and not labeling blame.

### Starting off

- Introductions, as necessary.
- The leader can briefly recap the tough situation.

### The dialogue

- Ask open ended questions for what could be done better.
- Ask if participants note any areas for improvement.
- Ask how colleagues are doing psychologically/emotionally.

### Record action items

- Select a person to be a scribe.
- Record ideas for QI.
- Record any items that are outside of the scope of the debrief.

### Ending the debrief

- Ask for any comments/feedback prior to ending.
- Provide a summary of the debrief, rounding out the talking points and action items.

### After the debrief

- Be familiar with resources available to staff who need additional assistance. For example, human resource (employee health) facilitated mental health outreach programs or social workers.
- It is critical that leaders of the group watch out for individuals at each level (attending, resident, nurse/technologist) who may have an extreme reaction to the event (e.g., a resident who makes a mistake and a patient directly dies) and guides them to appropriate support.
- Assign someone (or task yourself) to convey action items to appropriate channels. A unit manager or lead technician could fill this role well.
- Provide peer support, as able.

### Asking for help

- This whole process may be outside of the comfort zone of an IR. Colleagues in other specialties such as palliative care, emergency medicine, critical care or psychiatry may be a helpful resource to call upon for assistance in leading a debrief. Consultation with these peers could be beneficial for assistance with a successful session until comfort or experience is gained.

### Tips for success

- If the conversation starts going off track, be prepared to redirect gently.
- Be prepared to gently remind people of timeline and talking limits.
- Be prepared for the possibility of conflict and have a general plan for addressing this.

Of note, routine Morbidity and Mortality conferences can be considered as a type of “cold” debrief. Similar considerations and concepts can be applied to take psychological safety into consideration of the parties involved for the most benefit from the debrief.

## Conclusion

Despite a paucity of specialty specific literature in Interventional Radiology, debriefing is an available technique allowing for involved parties to partake in learning and QI as well as provide psychological and emotional support to a team. Additionally, debriefs can positively affect team performance.

IRs should familiarize themselves with the process and/or seek mentorship in leading a debrief. It is yet another tool in the vast tool chest of an IR to lead a team in exceptional patient care.

## Data Availability

Not applicable.

## Abbreviations

IR: Interventional Radiologist
AE: Adverse Event
CISD: Critical Incident Stress Debriefing
QI: Quality Improvement
AAR: After Action Review
